# Marijuana-induced Coronary Vasospasm with Persistent Inter-coronary Connection: A Case Report and Review of Literature

**DOI:** 10.7759/cureus.4799

**Published:** 2019-06-01

**Authors:** Janani Baskaran, Mahesh Anantha Narayanan, Jasmine Vakhshoorzadeh, Aiza Ahmad, Stefan Bertog

**Affiliations:** 1 Internal Medicine, Creighton University, Omaha, USA; 2 Cardiology, University of Minnesota, Minneapolis, USA

**Keywords:** prinzmetal angina, vasospastic angina, coronary vasospasm, marijuana, acute myocardial infarction, coronary arcade, coronary circulation, coronary vessel anomalies

## Abstract

Coronary vasospasm is a well-known entity causing acute chest syndrome and can lead to myocardial infarction, ventricular arrhythmias, and even sudden cardiac death. While there are extensive case series showing the association of coronary vasospasm with cocaine, studies reporting marijuana-induced coronary vasospasm are limited in number. We herein present a case of coronary vasospasm in a middle-aged African-American male who presented to the emergency department after an episode of syncope. His urine drug screen was positive only for marijuana. He had a transient elevation of ST segments on his EKG with concomitant wall motion abnormalities on echocardiogram and was later found to have vasospasm of coronary arteries on coronary angiogram without any evidence of focal atherosclerotic disease. Another interesting finding was the persistent inter-coronary communication or coronary arcade connecting the left circumflex artery to the right coronary artery. There was bi-directional flow through the inter-coronary communication and hence, we believe this communication prevented our patient from experiencing acute chest symptoms or myocardial infarction. It is important for the clinicians to recognize the association of marijuana with coronary vasospasm. At the same time, these patients should be treated as acute coronary syndromes until proven otherwise by ischemia evaluation.

## Introduction

Coronary vasospasm, also known as Prinzmetal angina, is a well-known entity that can lead to myocardial infarction, arrhythmias, and even sudden cardiac death [1]. While the association between cocaine and vasospastic angina is well established, the association of marijuana with coronary vasospasm has not been reported frequently [2]. We hereby present a case of an adult male who presented with syncope and ST-elevation on EKG without angina and was found to have coronary vasospasm secondary to marijuana ingestion. Interestingly, the patient had a large inter-coronary connection (ICC) between the right and left coronary arteries with a bidirectional flow, which we believe prevented significant myocardial infarction.

## Case presentation

A 60-year old African-American male with hypertension, hyperlipidemia, diabetes, and no previous cardiac history presented to the emergency room (ER) after a syncopal event. The patient did not require cardiopulmonary resuscitation or airway protection. In the ER, he denied any shortness of breath, chest pain, or palpitations prior to the syncopal event. He had undergone right knee arthroplasty two weeks ago and was taking hydrochlorothiazide and aspirin at home. He had quit cigarette smoking three years ago but was actively using marijuana.

**Figure 1:**
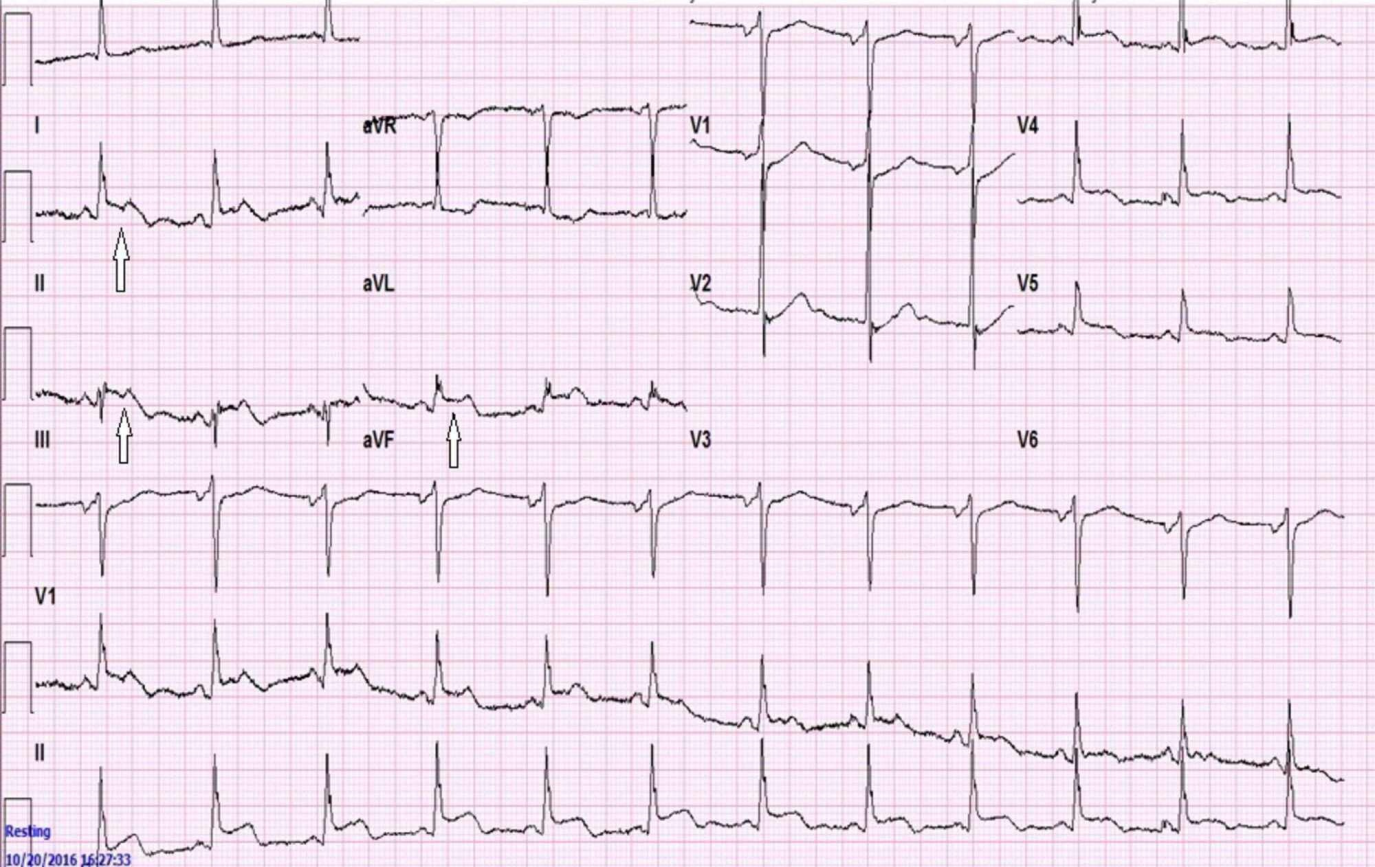
EKG showing ST-segment elevation in the inferior leads (*arrows point to ST-elevation in the inferior leads)

At presentation, his vitals were blood pressure 119/79 mm Hg, pulse 90 beats per minute, and temperature 97.9 Fahrenheit, and he was saturating at 99% on room air. Initial EKG obtained in the ER showed sinus rhythm with 1-mm ST-elevations in the inferior leads without reciprocal changes (Figure 1).

**Figure 2:**
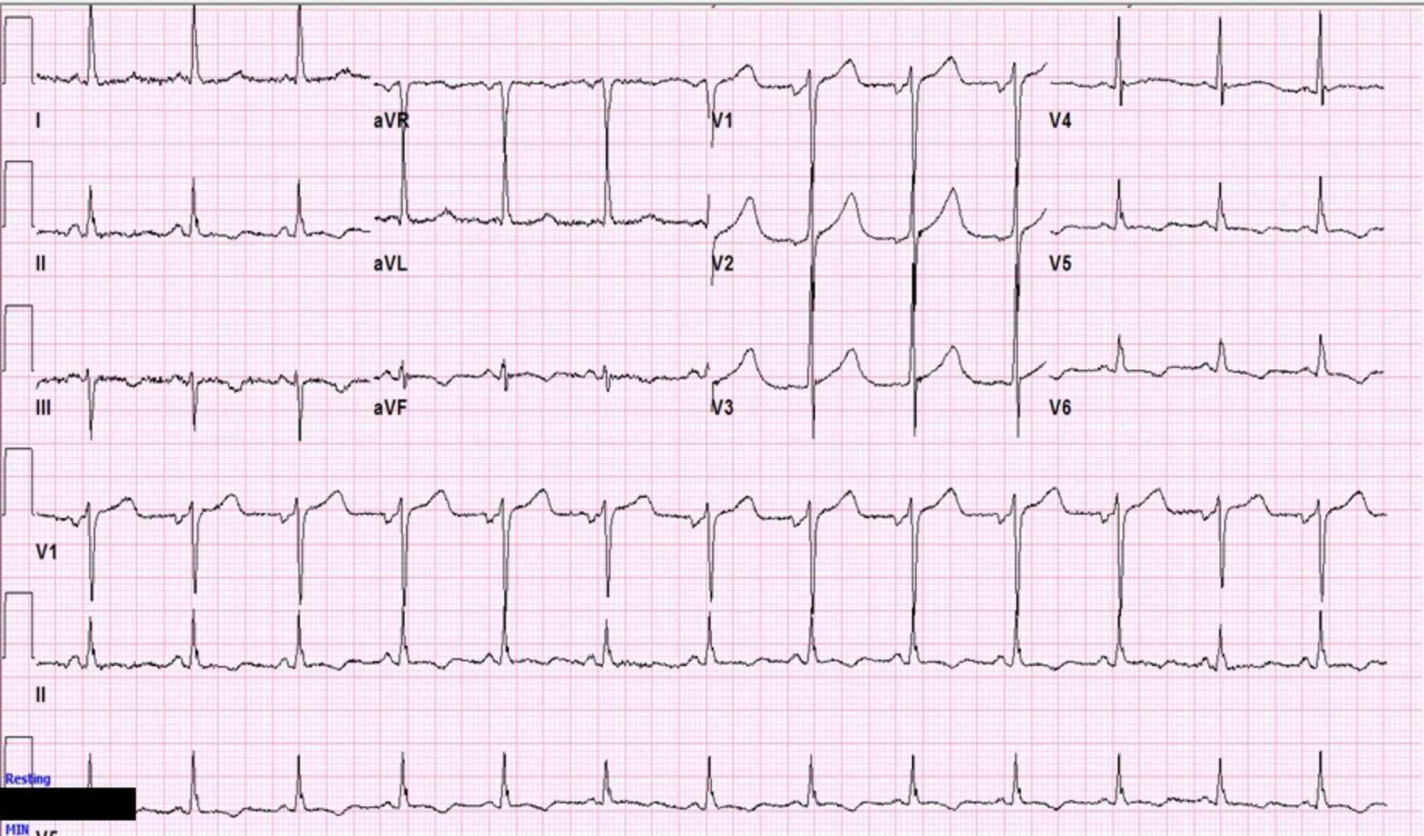
Repeat EKG showing resolution of ST-segment elevations in the inferior leads

Repeat EKG obtained 10 minutes later showed complete resolution of the ST segment elevation (Figure 2).

Laboratory workup revealed normal hemogram and metabolic panel, negative troponin, and a positive D-dimer of 2653 ng/ml. His urine drug screen was positive for marijuana and negative for all other drugs on the panel, including cocaine. With elevated D-dimer in the setting of recent orthopedic surgery, a CT angiogram of the chest was done which did not reveal pulmonary embolism or aortic dissection. A bedside echocardiogram in the ER showed basal inferolateral wall motion abnormality with the preserved ejection fraction of 55% to 60% (Video 1).

**Video 1:** Transthoracic echocardiogram showing basal inferolateral wall motion abnormality

**Figure 3:**
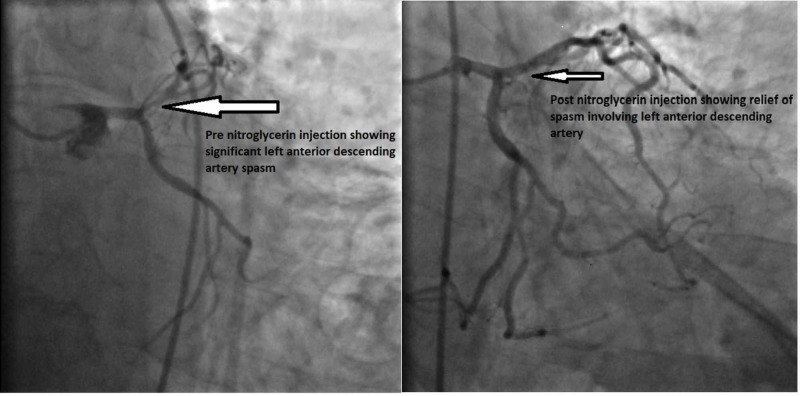
Angiogram images of LAD showing relief of vasospasm post nitroglycerin injection LAD, left anterior descending artery

Following this, the patient was taken directly to the cardiac catheterization lab. His left main coronary artery was found to be normal, but the left anterior descending artery (LAD) had severe 80% proximal stenosis that resolved with intracoronary nitroglycerine (Figure 3).

The left circumflex vessel (LCx) was normal but had a large ICC filling the right coronary artery (RCA). The RCA had a 70% proximal stenosis which was also relieved with nitroglycerine. Injection of RCA filled the LCx through the arcade (the same arcade which filled RCA during LCx injection; Video 2).

**Video 2:** Coronary angiogram showing selective right coronary contrast injection after relief of vasospasm by intracoronary nitroglycerine Note the large inter-coronary connection extending between the right coronary artery (right side of the screen) and the left circumflex artery (where the inter-coronary connection ends).

**Figure 4:**
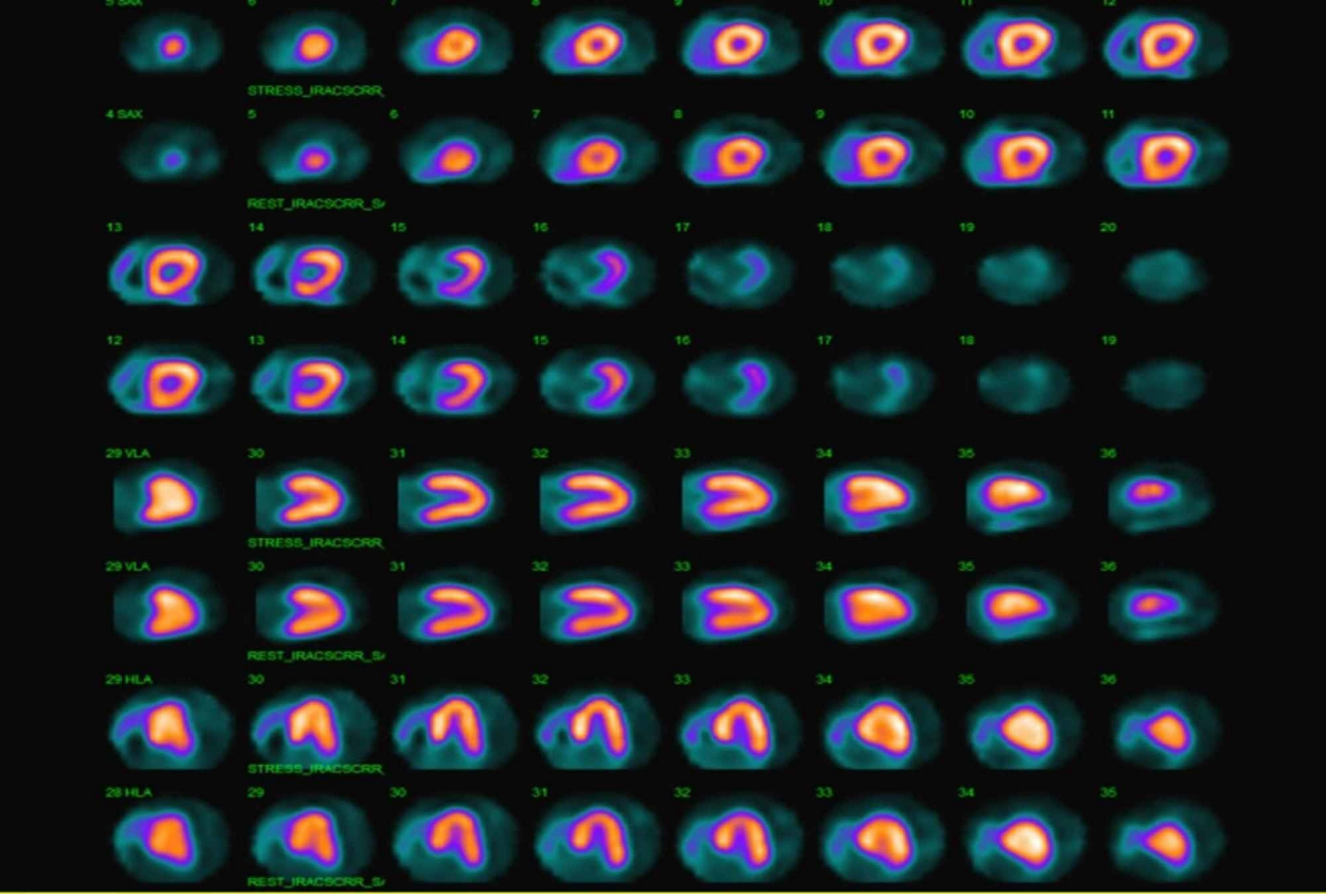
Pharmacological stress test showing no evidence of fixed or reversible ischemia

The patient underwent a pharmacological nuclear stress test to evaluate for exercise-induced vasospasm, which showed no fixed or reversible ischemia (Figure 4).

The patient was discharged home later that day on long-acting nitrate and calcium channel blocker after extensive counseling on the importance of quitting marijuana.

## Discussion

Marijuana has been shown to be the most commonly abused psychoactive drug in the United States. According to the National Survey on Drug Use and Health 2013, 7.5% (~20 million) of the U.S. general population >12 years of age reported using marijuana [3].

Drug-induced vasospasm is an important consideration in younger individuals with no prior cardiac history. Cocaine, amphetamines, and Methylenedioxy​methamphetamine (MDMA) commonly called 'Ecstasy' are the recreational drugs most commonly associated with vasospasm. While marijuana is well known to cause cardiac effects such as dose-dependent tachycardia, hypertension, and an increased risk of arrhythmias, myocardial infarction or vasospasm has rarely been described. In an epidemiological study to identify triggers of non-fatal myocardial infarction, marijuana was shown to have a low population attributable factor of 0.8 (95% CI 0.38% to 1.67%); in comparison, cocaine use had an odds ratio of 24 [4]. Mittleman et al. showed an elevated risk of myocardial infarction at 4.8 times over baseline (95% CI, 2.4 to 9.5) in the first 60 minutes following cannabis use; this risk rapidly decreased in the hours thereafter [5].

Different mechanisms have been proposed to explain the deleterious effects of cannabis on the heart. The pathophysiology is thought to be inadequate or no-flow in the coronary vessels, high catecholamine release resulting in increased oxygen demand in the setting of vasoconstriction and coronary vasospasm [6]. Cannabinoid receptors (CB) in humans are of two types: CB1 (pro-atherogenic) and CB2 (anti-atherogenic). There was an upregulation of macrophage CB1 receptor and increased reactive oxygen species (ROS) generation in advanced atheromas of patients with unstable angina compared with those with stable angina [7]. Rimonabant is a CB1 receptor antagonist that was shown to decrease the total atheroma volume in the STRADIVARIUS trial (Strategy To Reduce Atherosclerosis Development InVolving Administration of Rimonabant - the Intravascular Ultrasound Study) [8]. Further trials are underway to evaluate the usefulness of rimonabant in coronary artery disease.

Coronary spasm most often occurs in angiographically normal arteries, although when spasm does occur in the presence of atherosclerotic plaque, outcomes have been shown to be worse, even in non-significant stenosis [9-10]. The coronary spasm can cause rupture of atherosclerotic plaque causing coronary thrombosis and myocardial infarction. In chronic stable angina, spasm can lead to myocardial ischemia [11]. The coronary spasm can have a variety of presentations and delay in diagnosis is not uncommon [12]. The median time to presentation is three months and, in some cases, may present with sudden MI or cardiac arrest [12]. In coronary vasospasm, there is transient ST elevation/depression in multiple leads and the EKG quickly returns to baseline as seen on the repeat EKG in our patient [13]. Provocation testing can be performed using ergonovine or acetylcholine [14]. Our patient had spontaneous coronary vasospasm during the angiogram and hence did not require any provocation testing.

Inter-coronary communication (ICC) or coronary arcade between two coronary arteries is a very rare anomaly with a prevalence of <0.05% [15]. These are large caliber connections and differ from collaterals. Typically, they are larger in diameter (>1 mm), extramural, and straight compared to collaterals that are small and tortuous. Histologically, these communicating arteries are similar to normal adult coronary arteries, and therefore, it is proposed that this anomaly results from mere persistence of a certain type of fetal pattern of coronary circulation [16]. The significance of ICC is unknown, but it has been shown to be protective of ischemia or infarction through bidirectional blood flow [17]. In certain cases where the blood flow is angiographically observed to be unidirectional, this inter-coronary communication can cause myocardial ischemia through coronary steal phenomena [18]. In the above-mentioned case, however, we hypothesized that two-way ICC between RCA and LCx was protective in preventing an acute infarction despite significant EKG changes and wall motion abnormality on echocardiogram.

## Conclusions

This case illustrates the importance of recognition of vasospasm in patients taking marijuana. With the legalization of marijuana in certain states, marijuana-related hospitalizations and ER visits are likely to increase. It is therefore important for clinicians to know the various effects of marijuana, especially potentially fatal ones like coronary vasospasm. Emergency physicians should consider this in the differential diagnosis of patients presenting with ischemic symptoms based on history and a urine drug screen positive for cannabinoids. As this is a diagnosis of exclusion, it is prudent to treat these patients as having an atherosclerotic coronary disease until proven otherwise.
